# Diagnostic difficulties in the diagnosis of high acute-phase proteins levels in a teenage drug addicted female patient

**DOI:** 10.1186/s12888-022-04135-3

**Published:** 2022-07-28

**Authors:** Helena Krakowczyk, Maria Szczepańska, Urszula Wróblowska-Grzonka, Katarzyna Gajewska-Ormińska, Katarzyna Ziora, Edyta Machura

**Affiliations:** 1 Department of Pediatrics, Faculty of Medical Sciences in Zabrze, Medical University of Silesia, Katowice, Poland; 2 Department of Child Health, District Hospital in Rydułtowy, Rydułtowy, Poland

**Keywords:** C-reactive proteins, Procalcitonin, Inflammation, Drug addiction

## Abstract

**Background:**

Youth drug addiction is a significant social and health problem. Symptoms of the disease include a number of neurological, gastrointestinal and cardiovascular disorders. Possible hormonal disorders and dysregulation of the immune system could also occur.

**Case presentation:**

We describe a case of a teenage patient with multiple diseases such as esophagitis, allergic disease, and numerous behavioral disorders leading to: self-injury of the body, suicide attempts by drugs overdosing, and experimentation with various psychoactive substances (morphine, amphetamine, methamphetamine, codeine). She was also diagnosed with bipolar disorder. A few hours before the admission to the ward, the patient had an intravenous injection of drugs. Toxicological tests confirmed the presence of amphetamine, ecstasy and opioids in the blood and urine. Laboratory tests revealed extremely increased inflammatory parameters, leucopenia, increased levels of IgG, IgA and IgE (total) immunoglobulins, low concentration of vitamin D. Bacteriological examinations were negative. General condition of the patient got better very quickly, antibiotic therapy was abandoned on the 4^th^ day. It was concluded that the cause of the elevated concentration of acute-phase proteins was most likely caused by intoxication with psychoactive drugs.

**Conclusions:**

The discussed case shows the difficulties of differential diagnosis in a teenage patient struggling with many diseases, who has been abusing drugs for several years. Increased inflammatory parameters in the form of an raised PCT, CRP, NLR, PLR values may be caused by many factors. In adolescents who frequently experiment with psychoactive substances, such cause of these disturbances should also be taken into account.

## Background

Youth drug addiction is a significant social and health problem. The World Health Organization defines addiction as a mental and physical state resulting from the interaction between a living organism and a chemical substance (alcohol, drugs, drugs), characterized by changes in behavior and other reactions, which include the need to take the substance continuously or periodically in order to experience its impact on the psyche or to avoid unpleasant symptoms accompanying the lack of substances. Adolescents are the group of people most prone to addiction. The critical age of initiation of drug use begins during the adolescent period. During this period, young people have a strong inclination toward experimentation, curiosity, susceptibility to peer pressure, rebellion against authority, and poor self-worth, which make such individuals vulnerable to drug abuse [1, 2]. Literature data confirmed that in adolescents, disturbed family relationships such as poor parental supervision, unstable family structure and improper contacts between peers, are predisposing factors for the development of addiction. Usually they coexist with the absence of the so-called protective factors: high self-esteem, religiosity, determination [3, 4]. Adolescent who abuse drugs are also reported to have higher rates of physical and mental illness and reduces overall health and well-being [1, 2].

We present below a case of a teenage patient with multiple diseases, in whom the elevated parameters of inflammation and high levels of antibodies resulting from the activation of the immune system were most likely related to the abuse of psychoactive substances.

## Case presentation

A 16.5-year-old girl was admitted urgently to the General Pediatric Ward of Independent Public Hospital No. 1 in Zabrze due to uncontrolled vomiting, severe headache and fever. A few hours before the admission to the ward, the patient had an intravenous injection of methamphetamine along with morphine, which also regularly happened in the weeks preceding the current hospitalization. The patient's family history was burdened with Hashimoto's disease in her mother, allergic conditions of her father and her 3 years younger brother. From the pre-school period, the patient has been diagnosed with recurrent respiratory tract infections, and from the age of 6 in the spring and summer period, symptoms of hypersensitivity to grass pollen in the form of allergic rhinitis and seasonal asthma have been observed.

At 11 years of age aseptic necrosis of the right tibial tuberosity was confirmed, and at 12 years of age adenoidectomy was performed.

At 13 years of age due to recurrent abdominal pain for 3 years, gastroenterological diagnostics was performed. Lactose and fructose intolerance, and celiac disease were excluded. Esophagitis was diagnosed in the gastroscopic examination corresponding to grade A in the Los Angeles Classification of reflux esophagitis and gastroprotective treatment was recommended from that time. In addition, since the age of 13, the girl has exhibited behavioral disorders, performed self-harm to the body. She was experimenting with various psychoactive substances (morphine, amphetamine, methamphetamine, codeine).

At 15 years of age she attempted suicide by overdosing of drugs, which coincided with a difficult family situation (parents' divorce). Due to the above-mentioned mental disorders and adaptation difficulties, in the last 2 years she was hospitalized twice in the Youth Psychiatric Ward, where she was diagnosed with bipolar disorder. Pharmacological treatment was continued since October 2020 with arypiprazol 15 mg in the morning, pregabalin 75 mg in the evening, quetiapine XR 400 mg in the evening. For a year, she has been under the outpatient care of the Addiction Treatment Center, but the therapy was not effective. Until now, the patient has not consented to therapy in an inpatient center. Legal steps have been taken to issue appropriate treatment decisions for the teenager.

On admission, the patient had a fever up to Celsius 40-degree and complained of generalized pain. In addition, deviations from the normal state included tachypnea, fine-wave tremors of the upper and lower limbs, skin hyperalgesia, tattoos covering the neck, limbs and trunk, as well as numerous fresh and old scars of self-injury, features of dehydration, tachycardia (heart rate-120–130 beats/min), abdominal skin tenderness and dry oral mucosa were recorded. Laboratory tests revealed significantly increased inflammatory parameters: C-reactive protein (CRP) -37.4 mg/l, procalcitonin (PCT) 50.46 ng/ml), leukopenia with lymphopenia, monocytosis and eosinopenia, hypocapnia in capillary blood gas tests, increased levels of immunoglobulin G, A (IgG, IgA) and total immunoglobulin E (IgE), low concentration of vitamin D (Table 1). Rapid SARS-CoV-2 antigen test was negative. While awaiting toxicological examination, parenteral hydration and empirical broad-spectral antibiotic therapy (cefuroxime) were used in the treatment. Toxicological tests confirmed the presence of amphetamines, ecstasy and opioids in blood and urine. On the second day of the patient's stay in the ward, a significant improvement in her general condition was observed and fever subsided. Neurological examination revealed no signs of focal CNS damage, meningeal symptoms were negative. The performed examinations: bacteriological (upper respiratory swabs, blood and urine cultures), thorax X-ray, ultrasound examination of the abdominal cavity, electrocardiography and echocardiographic examination were normal, therefore antibiotic therapy was abandoned on the 4^th^ day. Psychological and psychiatric examinations, carried out during hospitalization confirmed behavioral disorders, substance abuse, bipolar disorder, and the patient was transferred to a psychiatric ward for further treatment. Ultimately, it was concluded that the elevated concentration of acute-phase proteins was most likely caused by intoxication with psychoactive drugs. After a month, the patient reported for periodic check-up at an allergy clinic. During the observation of the patient, and due to the mother's interview data about not taking any therapeutic measures in her daughter, a suspicion of further abuse of psychoactive substances was raised, which was confirmed in a narcotic test (present—tetrahydrocannabinol, amphetamine, ecstasy). Routine laboratory tests performed at that time, except for the elevated CRP (17.38 mg / l), neutrophils count to lymphocytes count ratio ( NLR) (3,41 ratio), platelets count to lymphocytes count ratio (PLR) (142,85 ratio) were normal.

**Table 1:** Parameters’ values of the patient during hospitalization

Parameter	Result	
	At the beginning of hospitalization	At the end of hospitalization
**Vital signs**		
HR/min RR mmHg Number of breath/min Temperature	120-130 b/min 90/67 26 39.9 degree C	70-80 b/min 100/90 12 36.3 degree C
**Laboratory parameters**		
C-reactive protein (mg/L) (0-5)	37.4	1.84
Procalcitonin (ng/mL) (0-0.5)	50.46	0.33
Total leukocyte count (cells/µl)	2.36	9.33
Differential count (%)/(cells/µl)		
Neutrophils	75.9/1.77	64/5.97
Lymphocytes	12.3/0.29	26.8/2.5
Monocytes	11/1.52	6.5/5.5
Eosynophils	0.8/0.02	2.7 /1.86
Platelets (cells/µl)	213	337
NLR (neutrophils count to lymphocytes count ratio) (1,30 +/-0,31)*	6,1	2,38
PLR (platelets count to lymphocytes count ratio) (88,95 +/- 24,6 )*	734,48	134,8
Gasometry:		
pH p CO2 mmHg p O2 mmHg HCO3 mmol/l BE mmol/l	7,424 30,3 57,1 19,9 -3,3	
IgA (g/l) (0.33-2.35)	3.49	
IgM (g/l) (0.36-1.98)	1.72	
IgG (g/l) (8.53-14.4)	13.33	
IgE (IU/ml) (<100)	498	
C3 (g/l) (0.9-1.8)	1	
C4 (g/l) (0.1-0.4)	0.15	
Serum Creatinine umol/L	70	71
Sodium (mEq/L)	142	138
Potassium (mEq/L)	4.04	3.98
Aspartate aminotransferase (IU/L) (0-40)	19.6/64.7	24.7
Alanine aminotransferase (IU/L) (0-41)	23.6/92.8	56.8
Creatine kinase (U/L)(10-200)	22	
Troponin (ng/ml)	<0.003	
Prothrombin time (11-16)	13.8	
APTT (28-40)	27.4	
d-Dimer ug/ml (0-0.5)	0.69	
Vit D3 ng/ml (30-50)	9.99	
TSH uIU/ml (0,27-4,2) fT4 ng/dl (0,93-1,7)	1,77 2,08	
Total cholesterol (<5 mmol/l)	3.48	
Cholesterol HDL (>1.3 mmol/l)	0.71	
Cholesterol LDL (<2.6 mmol/l)	1.54	
Triglicerides (<1.7 mmol/l)	2.71	
**Microbiological testing**		
Blood culture	Negative	
Urine culture	Negative	
Rapid SARS-CoV-2 antigen test	Negative	

*by Cicek [19]

## Discussion and conclusions

In the clinical case we have described it was not easy to establish the diagnosis at the stage of initial medical and physical examination. The patient's multiple morbidity and mental state made it impossible to obtain precise information on the events preceding the current illness. The symptoms presented by the patient, such as fever, tachycardia, tachypnoe, leukopenia and hypocapnia in blood gasometry, met the criteria of the SIRS. It most often develops as a result of severe bacterial, viral and fungal infections, less often under the influence of mechanical injuries, prolonged surgical procedures, burns, pancreatitis, heat stroke as well as under the influence of chemicals, including drugs. SIRS symptoms are the result of excessive release of pro-inflammatory cytokines (TNF-a, Il-1, IL-6), acting locally and systemically. These are responsible, among others for the increase in temperature and the synthesis of acute phase proteins (CRP, PCT, fibrinogen) [5]. As is known, PCT and CRP are important biomarkers of bacterial sepsis, so identifying SIRS from a cause other than sepsis at the initial diagnostic stage is a difficult task, as we experienced in our case as well. Procalcitonin, like the CRP participates not only in the non-specific response of the organism to infectious stimuli, but its concentration increases, among others in extensive tissue damage during injuries, surgery and burns, in acute pancreatitis, and myocardial necrosis [6–9]. Under physiological conditions PCT is an intermediate product of the synthesis calcitonin produced by the thyroid gland in C cells, CRP is produced by hepatocytes in trace amounts. CRP determined in the low range of its concentrations by methods of high analytical sensitivity (hsCRP) reflects low grade inflammation, which is attributed to a significant role in the pathogenesis of atherosclerosis. The usefulness of the hsCRP test was also demonstrated in addicted adults in the 18–48 age group, finding significantly higher hs-CRP in the addicted group as compared to healthy controls. Therefore, it has been suggested that chronic opiate abuse leads to constant stimulation of atherogenesis with the participation of CRP, which takes part in all stages of formation, remodeling and destruction of atherosclerotic plaques, resulting in many health consequences described in drug addicts [10]. As mentioned above, an increased concentration of CRP was also found in our patient during the outpatient control, probably resulting from constant drug abuse, but also probably from disturbed energy homeostasis in the patient, as evidenced by: increased body mass index (BMI) (25.6 kg/m^2^), significantly exceeding the norm, high serum concentration of triglycerides and low level of high-density lipoprotein (HDL) cholesterol. All these abnormalities may be a significant factor in cardiovascular events in this patient.

Due to the symptoms presented at admission, such as fever, vomiting, headaches, and very high inflammatory parameters, the presence of CNS infection was considered as the cause of the disorders. However, due to the lack of any abnormalities in the neurological examination, the suspicion of intoxication with psychoactive substances, and then a quick improvement of the general condition with a dynamic decrease in inflammatory parameters, lumbar puncture and brain imaging were finally abandoned.

The symptoms of tachycardia, fever, tachypnoe, nausea, vomiting, limb muscle tremors, dryness of mucous membranes and skin, described on admission, were the symptoms of intravenous administration of a large dose of amphetamine, responsible for the stimulation of the sympathetic part of the autonomic nervous system. On the other hand, no symptoms typical of amphetamine poisoning, such as pupil dilation, blood pressure increase, psychomotor agitation, panic attacks, were observed. Perhaps this was due to the simultaneous intake of morphine, which has an antagonistic effect in relation to amphetamines. It stimulates the parasympathetic autonomic nervous system, especially its centers in the brain and spine. It has a euphoric effect, causes drowsiness up to coma, respiratory disorders up to apnea, bradycardia, hypotension, arrhythmias and impaired conduction of the heart stimulus system, urinary retention, pupil constriction [11, 12]. There are case reports [13–17] of extremely high levels of inflammatory markers (PCT levels up to a thousand times the normal limit) without concomitant bacterial infection in adult drug addicts after an overdose of amphetamines and opioids. In these patients, apart from hyperthermia and tachycardia, significant psychomotor agitation requiring administration of high doses of neuroleptic and antipsychotic drugs was observed, which resulted in respiratory disturbances and the need to introduce emergency breathing. In addition, transient hepatic dysfunction, rhabdomyolysis and acute kidney injury have been reported. In our patient, kidney tests, muscle and cardiac enzymes levels were normal, only a temporary increase in transaminases was observed. As in the described cases, after the exclusion of bacterial infection, antibiotic therapy was abandoned after 4 days.

In people using psychoactive substances, disorders in the immune system are also observed, which predispose to recurrent infections. Few studies showed a significant increase in serum IgA, IgM, IgG or IgG3, IgG4 concentration and the elevation of percentage of B lymphocytes subpopulation, lymphopenia and monocytosis in a group of addicted young people, similarly to our patient [18, 19]. As mentioned, the patient was diagnosed with bipolar disorder. It is believed that imbalances between pro and anti-inflammatory cytokines play a role in the pathogenesis of the disease. High levels of pro-inflammatory cytokines Il-6, TNF alpha, C-reactive protein, especially during the manic episode, and to a lesser extent in the depressive phase, have been documented [19]. Incresed values of NLR and PLR are considered to be an indicator of increased inflammation. NLR and PLR indices were found to be elevated in heroin dependence and psychiatric disorders such as schizophrenia, bipolar disorder and depression [20]. High values of NLR and PLR were found also in our patient upon admission—several hours after intravenous amphetamine intake. They were gradually reduced in the following days of hospitalization, but they were higher than values observed in healthy adolescents at the same age group [19]. Next control laboratory tests performed one month after the initial hospitalization showed a re-increase of these indicators, which could be attributed to the continuous use of psychoactive substances. Additionally, the patient was confirmed with significant hypovitaminosis D (9.99 ng / ml), which, according to the literature, may be associated with mood disorders, depression and bipolar disorder [21, 22]. In addition, low vitamin D3 levels are a predictor of suicide attempts. Determination of the concentration of Vit D3 and treatment of its possible deficiency may therefore be beneficial in the treatment of depressive disorders [23]

In the absence of one recommended single standard of care in patients abusing drugs in the event of the appearance of high levels of acute –phase proteins, the only legitimate way to proceed is the exclusion of the other possible causes of this increase, such as infection, in particular sepsis, psychiatric disorders, and others. The only gold standard for dealing with an acute patient after intoxication with psychoactive substance is a detailed interview, thorough examination, and diagnosis by exclusion of the organic causes of this condition.

Since infections were excluded and the other comorbidities like bipolar disorder and allergic disease were well controlled, the elevation of acute phase proteins along with clinical signs, of methamphetamine use—like fever, tachycardia, dry skin—and the decrease in those levels after cessation of methamphetamine use suggested that the elevation of acute phase proteins was probably due to drug abuse.

The discussed case displays the difficulties of differential diagnosis in a teenage patient struggling with many diseases, who has been abusing drugs for several years, remains in an ineffective family care system, which results in the lack of effectiveness of the undertaken therapies and further destruction of the girl’s health condition, both in the mental and somatic aspects. Increased inflammatory parameters described by the rise in PCT, CRP, NLR, PLR may be caused by many factors. In adolescents who frequently experiment with psychoactive substances, such cause of the increase of these parameters should also be taken into account.

## Data Availability

Data sharing is not applicable to this article as no datasets were generated or analysed.

## Abbreviations

CRP: C-reactive proteins
PCT: Procalcitonin
IgG: Immunoglobulin G
IgA: Immunoglobulin A
IgE: Immunoglobulin E
NLR: Neutrophils count to lymphocytes count ratio
PLR: Platelets count to lymphocytes count ratio
SIRS: Systemic inflammatory response syndrome
TNF-a: Tumour Necrosis Factor alpha
Il-1: Interleukin 1
IL-6: Interleukin 6
hsCRP: High sensitivity C-reactive protein
BMI: Body mass index
HDL: High-density lipoprotein cholesterol
